# Staphylococcal Toxic Shock Syndrome 2000–2006: Epidemiology, Clinical Features, and Molecular Characteristics

**DOI:** 10.1371/journal.pone.0022997

**Published:** 2011-08-10

**Authors:** Aaron S. DeVries, Lindsey Lesher, Patrick M. Schlievert, Tyson Rogers, Lourdes G. Villaume, Richard Danila, Ruth Lynfield

**Affiliations:** 1 Minnesota Department of Health, Emerging Infections Program, St. Paul, Minnesota, United States of America; 2 Department of Microbiology, University of Minnesota, Minneapolis, Minnesota, United States of America; 3 Department of Medicine, University of Minnesota, Minneapolis, Minnesota, United States of America; University of California, San Francisco, United States of America

## Abstract

**Introduction:**

Circulating strains of *Staphylococcus aureus* (SA) have changed in the last 30 years including the emergence of community-associated methicillin-resistant SA (MRSA). A report suggested staphylococcal toxic shock syndrome (TSS) was increasing over 2000–2003. The last population-based assessment of TSS was 1986.

**Methods:**

Population-based active surveillance for TSS meeting the CDC definition using ICD-9 codes was conducted in the Minneapolis-St. Paul area (population 2,642,056) from 2000–2006. Medical records of potential cases were reviewed for case criteria, antimicrobial susceptibility, risk factors, and outcome. Superantigen PCR testing and PFGE were performed on available isolates from probable and confirmed cases.

**Results:**

Of 7,491 hospitalizations that received one of the ICD-9 study codes, 61 TSS cases (33 menstrual, 28 non-menstrual) were identified. The average annual incidence per 100,000 of all, menstrual, and non-menstrual TSS was 0.52 (95% CI, 0.32–0.77), 0.69 (0.39–1.16), and 0.32 (0.12–0.67), respectively. Women 13–24 years had the highest incidence at 1.41 (0.63–2.61). No increase in incidence was observed from 2000–2006. MRSA was isolated in 1 menstrual and 3 non-menstrual cases (7% of TSS cases); 1 isolate was USA400. The superantigen gene *tst-1* was identified in 20 (80%) of isolates and was more common in menstrual compared to non-menstrual isolates (89% vs. 50%, p = 0.07). Superantigen genes *sea*, *seb* and *sec* were found more frequently among non-menstrual compared to menstrual isolates [100% vs 25% (p = 0.4), 60% vs 0% (p<0.01), and 25% vs 13% (p = 0.5), respectively].

**Discussion:**

TSS incidence remained stable across our surveillance period of 2000–2006 and compared to past population-based estimates in the 1980s. MRSA accounted for a small percentage of TSS cases. *tst-1* continues to be the superantigen associated with the majority of menstrual cases. The CDC case definition identifies the most severe cases and has been consistently used but likely results in a substantial underestimation of the total TSS disease burden.

## Introduction

A syndrome of fever, myalgias, sore throat, edema, scarlitiniform rash, and desquamation associated with *Staphylococcus aureus* (SA) infection was first described in 1927, and in 1978 Todd et al. coined the term staphylococcal toxic shock syndrome (TSS) [1]–[2]. By 1980, young menstruating women using high absorbency tampons were identified as a high risk group, with cases also observed in men and non-menstruating women [3]–[4]. As the pathogenesis was better understood, it became clear that SA toxins called superantigens in conjunction with host susceptibility from the absence of anti-superantigen antibodies were risk factors for the development of TSS [5]–[6].

The estimated incidence of TSS in 1980 among young menstruating women was 13.7 per 100,000 persons [7]. Following multiple public health interventions including removal of highly absorbent tampons and messages regarding proper use of tampons, the number of cases declined sharply. By 1986 the rates of menstrual and non-menstrual TSS cases were 1 and 0.3 per 100,000, respectively [8]–[9]. In 1986, the overall case fatality rate was estimated to be 4%, with young women as the highest risk group (median age, 25 years) [9]. In the early 1980s long-term morbidity was observed in up to 90% of TSS cases, with 20% experiencing recurrent episodes, 50% having long-term memory loss and abnormal EEG findings, and 23% having recurrent syncope or cardiomyopathy [10]–[11]. Since 1986, there has not been population-based active surveillance to assess the incidence or disease burden of TSS.

Many strains of SA are known to carry genes for superantigens including toxic shock syndrome toxin–1 (*tst-1*), the causative superantigen in most TSS cases [12]–[14]. Prevalent strains of SA are in constant flux. Over the past 15 years, community-associated methicillin-resistant SA (CAMRSA) strains, most notably USA300 and USA400, have emerged as predominant causes of skin and soft tissue infection in many geographic regions of the United States [15]–[16]. Skin and soft tissue infections are a common primary site of non-menstrual TSS [10], [17]. Historically, children and adolescents had the highest prevalence of CAMRSA colonization and the highest incidence of CAMRSA infection, although more recently CAMRSA has been increasing in other age and risk groups such as hospitalized patients [18]–[20]. This is relevant because children have lower frequencies of protective anti-superantigen antibodies and are at greater risk to TSS if exposed to superantigen-producing SA strains [21]. The repertoire of superantigen genes produced by CAMRSA strains that are known to cause TSS is limited. The superantigen genes for staphylococcal enterotoxins A (*sea*), B (*seb*) or C (*sec*) have been described in USA400 [22]. *seb* has been associated with non-menstrual TSS cases [12], [23]–[25]. However, neither USA300 nor USA400 have been described to carry *tst-1* [13], [25]–[26].

The current passive surveillance system for TSS is limited given the complexity of the clinical diagnosis and lack of a single diagnostic test. It is unclear whether the continually evolving epidemiology of circulating SA strains is leading to a change in the incidence of TSS cases. A report of a rapid increase in the number of SA isolates from patients with TSS submitted for superantigen testing during 2000–2003 to a Minneapolis-St. Paul metropolitan area reference laboratory suggested that rates of disease may be on the rise [27]. Given the possibility of an increase in the incidence of TSS, particularly among young persons in association with changes in SA epidemiology, we began active, population-based surveillance to identify the current incidence of TSS in the Minneapolis-St. Paul metropolitan area.

## Methods

Cases of staphylococcal TSS are reportable to the Minnesota Department of Health (MDH) as required by the Minnesota Communicable Disease Rule. This study was reviewed and approved by the Institutional Review Board as exempt research without requirement for informed consent as this was review of existing data. The catchment area included the 7 counties of the Minneapolis-St. Paul (MSP) metropolitan area (Anoka, Carver, Dakota, Hennepin, Ramsey, Scott, and Washington), with a combined population of 2,642,056 (2000 U.S. Census). This area encompasses 50% of the total Minnesota population and 24 acute care hospitals from urban, suburban, and rural areas. There was no change in the catchment area across both surveillance periods (2000–2003 and 2004–2006).

### Surveillance Period 2000–2003

ICD-9 codes were used to identify potential cases of TSS. A list of all inpatient hospitalizations discharged between January 1, 2000 and December 31, 2003 that received at least one of the study codes was requested from all 24 hospitals in the MSP metropolitan area. All hospitals provided a list. Every hospitalization that received the specific ICD-9 code for TSS (040.82 or 040.89) was reviewed (Table 1). For hospitalizations that received at least one of the other ICD-9 codes (038.11, 038.19, 038.9, 785.50, 785.59 or 785.52), that were non-specific for TSS, a 20% random sample from within each hospital was reviewed. The medical records of all selected hospitalizations were reviewed for the Centers for Disease Control and Prevention (CDC) TSS case definition criteria (Table 2) [28] and other pertinent epidemiologic and clinical information. From 2000–2003 potential cases were also sought from Minnesota death certificates receiving the ICD-10 code for toxic shock syndrome (A48.3) and from cases reported to the Minnesota Unexplained Critical Illness and Death of Possible Infectious Etiology project (UNEX) [29]. Methods for surveillance from 2000–2003 were previously published [30].

**Table 1:** ICD-9 Study Codes Utilized for TSS Case Ascertainment.

ICD-9 Code	Associated Diagnosis
**Specific toxic shock syndrome code**	
040.89 or 040.82*	Toxic shock syndrome
**Non-specific toxic shock syndrome codes**	
038.11	*Staphylococcus aureus* septicemia
038.19†	Other staphylococcal septicemia
038.9	Unspecified septicemia
785.50	Shock without mention of trauma
785.59 or 785.52*	Sepsis

*The ICD-9 code number assigned to “toxic shock syndrome” changed from 040.89 to 040.82 on October 1, 2002, and the code number assigned to “sepsis” changed from 785.59 to 785.52 on October 1, 2003. While the numeric codes changed, their associated diagnoses remained unchanged and are considered mutually exclusive.

† Code eliminated after interim analysis.

**Table 2:** Surveillance Case Definition of TSS [28].

Clinical Criteria
1. *Fever*: temperature ≥38.9°C (102.0°F).
2. *Rash*: diffuse macular erythroderma.
3. *Desquamation*: 1–2 weeks after onset of illness, particularly on the palms and soles.
4. *Hypotension*: systolic blood pressure (BP) ≤90 mmHg for adults or less than fifth percentile by age for children aged <16 years; orthostatic drop in diastolic blood pressure ≥15 mmHg from lying to sitting, orthostatic syncope, or orthostatic dizziness.
5. *Multisystem involvement* (three or more of the following):
• Gastrointestinal: vomiting or diarrhea at onset of illness.
• Muscular: severe myalgia or creatine phosphokinase (CPK) level at least twice the upper limit of normal (ULN).
• Mucous membrane: vaginal, oropharyngeal, or conjunctival hyperemia.
• Renal: blood urea nitrogen (BUN) or creatinine (Cr) at least twice ULN for laboratory or urinary sediment with pyuria (≥5 white blood cells [WBC] per high-power field) in the absence of urinary tract infection.
• Hepatic: total bilirubin (T.Bili), alanine aminotransferase enzyme (ALT), or asparate aminotransferase enzyme (AST) levels at least twice ULN for laboratory.
• Hematologic: platelets ≤100×109/L.
• Central nervous system: disorientation or alterations in consciousness without focal neurologic signs when fever and hypotension are absent.
Laboratory criteria
6. Negative results on the following tests, if obtained:
• Blood, throat, or cerebrospinal fluid cultures (blood culture may be positive for *Staphylococcus aureus*).
• Rise in titer to Rocky Mountain spotted fever, leptospirosis, or measles.
Case classification
*Probable case*: meets the laboratory criteria and in which four of the five clinical findings described above are present.
*Confirmed case*: meets the laboratory criteria and in which all five of the clinical findings described above are present, including desquamation, unless the patient dies before desquamation occurs.

Five experienced record abstractors received initial and ongoing training in data abstraction using a Minnesota Department of Health (MDH)-developed eight-page TSS case report form. TSS cases were classified as menstrual if (i) the onset of symptoms was during documented dates of menstruation or (ii), in the absence of documented dates of menstruation, a woman was between the ages of 13 and 54 years *and* a vaginal culture was positive for SA. All other TSS cases were classified as non-menstural.

### Surveillance Period 2004–2006

Based on preliminary analysis of 2000–2003, the TSS-specific code identified cases with higher sensitivity and specificity compared to other codes and therefore only the TSS-specific code was used for surveillance period of 2004–2006 [30]. A list of all inpatient hospitalizations discharged between January 1, 2004 and December 31, 2006 that received the TSS-specific code was requested from all 24 hospitals in the MSP metropolitan area; all hospitals complied. All hospitalizations were reviewed using the same methodology as used during the surveillance period 2000–2003.

### Population Denominator Calculations

Population estimates were taken from the 2000 US Census and the inter-Census estimate for 2005. Assuming a linear rate of growth, an average of 0.8% increase in population per year was used for denominator calculations over the years 2000–2006. An average population was used to calculate incidence over the 4-year period from 2000–2003. For the estimated incidence of all TSS and non-menstrual TSS cases, the total population was used as the denominator. For menstrual cases, the denominator was females aged 13–55 years, as this was the approximate age distribution among our menstrual cases.

### Statistical Analysis

Cases of TSS with a home zip code outside the MSP area were included in the descriptive analysis of cases but were excluded from incidence estimations, as referral patterns strongly favor cases with home zip codes in the MSP area to be hospitalized in the MSP area (based on culture-confirmed invasive bacterial infections, Active Bacterial Core surveillance [31] in MN. For descriptive analysis, Student *t*-tests and Fisher's Exact Chi-square tests were used for continuous and categorical variables, respectively.

The average annual incidence was estimated for 2000–2003 as this was the period of most complete case ascertainment. In order to accommodate separate sampling frequencies, the TSS incidence rates for 2000–2003 were separately estimated among (i) the hospitalizations assigned a TSS-specific code and (ii) the hospitalizations assigned TSS-non-specific codes. These two incidence rates were summed, and the overall incidence rate was estimated by WinBugs version 1.4 software with Bayesian statistical methods and Poisson regression. Bayesian methods were chosen to estimate incidence rates as they can generate confidence intervals while allowing for the different rates of sampling of TSS-specific code and the TSS-non-specific codes. Bayesian statistics also permitted the evaluation of differences in incidence rates between age groups and across time. Frequentist Poisson regressions were performed using SAS 9.2 to corroborate Bayesian analysis and to calculate P-values for test of trend.

To assess a change in incidence over the years 2000–2006 only those cases identified using the TSS specific code were used. Frequentist Poisson regression was used to calculate P-values for test of trend across time.

### Characterization of isolates

All available SA isolates from confirmed or probable cases were collected. Isolates were tested by PCR for presence of superantigen genes *tst-1*, *sea*, *seb*, and *sec* [32]. Molecular typing of a limited number of isolates was performed by pulsed field gel electrophoresis (PFGE) of genomic DNA after restriction endonuclease digestion with Sma1. PFGE patterns were compared using Bionumerics Software34 and the Dice coefficient. USA clonal groups were determined by PFGE per SA PFGE genotype nomenclature [33]. Antimicrobial susceptibility testing was performed by clinical laboratories.

## Results

### Case Identification

**Figure 1:**
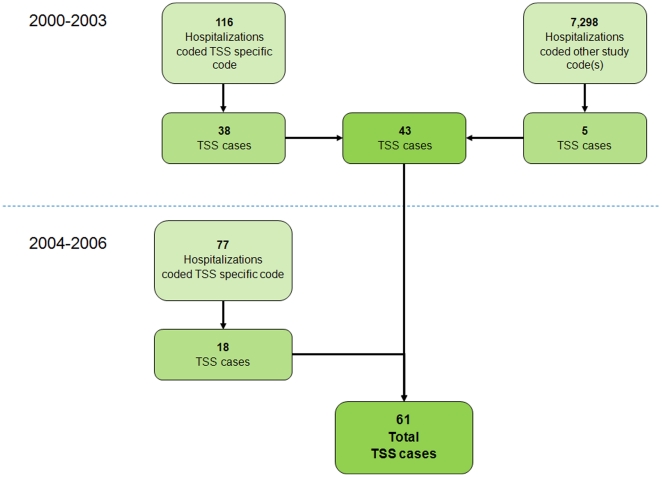
Flow diagram of TSS case ascertainment. From 2000–2003 TSS cases were identified from multiple data sources including ICD-9 hospital discharge codes, cases reported to the Minnesota Unexplained Critical Illness and Death of Possible Infectious Etiology project (UNEX), and death certificate data using ICD-10 code A48.3. From 2004–2006 TSS cases were identified from only the TSS-specific ICD-9 hospital discharge code.

From January 1, 2000 to December 31, 2003, we identified 7,414 hospitalizations with at least one study code and 43 probable or confirmed TSS cases, all from ICD-9 code surveillance (Figure 1). Cases were identified at 14 of the 24 hospitals, with 89% of cases at five hospitals. From 2000–2003, no additional cases were identified from 9 deaths coded with the ICD-10 code for TSS and from the 153 reported UNEX cases. Among the 43 cases from 2000–2003, the TSS-specific code was more likely to be used on menstrual cases [22/23, (96%)] compared to non-menstrual cases [16/20, (80%), p = 0.17]. From January 1, 2004 to December 31, 2006, an additional 77 hospitalizations receiving the TSS-specific code were identified (Figure 1) of which 18 were probable or confirmed TSS cases. The total number of TSS cases identified over years 2000–2006 was 61.

### Clinical Characteristics

Among the 61 TSS cases, the median age was 21.4 years (range, 1.4–81.0). The median age was 34.8 years (range, 4.5–81) for the 13 males and 18.5 years (range, 1.4–76.5) for the 48 females (male vs. female cases, p = 0.05). There were 33 menstrual and 28 non-menstrual TSS cases, with menstrual cases trending toward younger age and fewer preexisting conditions (Table 3). There were minimal differences in clinical presentation between menstrual and non-menstrual cases.

**Table 3:** Description of Toxic Shock Syndrome Cases.

	All Cases	Menstrual	Non-menstrual	
Characteristics	n = 61 (Range or % Total)	n = 33 (Range or % Total)	n = 28 (Range or % Total)	p value*
Median age (yr)	21.4 (1.4–81.0)	17.9 (12.4–52.6)	26.3 (1.4–81.0)	0.12
Female sex	48 (79%)	33 (100%)	15 (54%)	<0.001
MSP area home zip code	50 (82%)	28 (85%)	22 (79%)	0.74
Median days from first symptom to hospitalization	2 (0–7)	2 (0–7)	2 (0–7)	0.65
One or more co-morbidities†	19 (31%)	7 (21%)	12 (43%)	0.10
All six criteria	12 (20%)	9 (27%)	3 (11%)	0.12
Temperature ≥38.9°C (102.0°F)	59 (97%)	32 (97%)	27 (96%)	1.00
Hypotension‡	57 (93%)	32 (97%)	25 (89%)	0.33
Rash consistent with erythroderma	60 (98%)	32 (97%)	28 (100%)	1.00
Desquamation	20 (33%)	12 (36%)	8 (29%)	0.59
Multisystem involvement§	60 (98%)	33 (100%)	27 (96%)	0.46
Any culture positive for SA	44 (72%)	28 (85%)	16 (57%)	0.02
Median days of hospitalization	6 (2–50)	5 (2–25)	11 (2–50)	<0.001
Deaths	1 (2%)	0 (0%)	1 (4%)	0.46

Abbreviations: yr, years; MSP, seven-county Minneapolis-St. Paul metropolitan area; SA, *Staphylococcus aureus*; MRSA Methicillin-Resistant SA; MDH, Minnesota Department of Health.

*Comparison of menstrual and non-mentstrual TSS cases. Student *t*-tests were used for continuous variables and Fisher's Exact Chi-Square tests were used for categorical variables.

† Co-morbidities defined as in the Active Bacterial Core surveillance project [31].

‡ Hypotension defined as orthostatic hypotension or syncope, or systolic BP≤90 mmHg if age ≥16 yr, ≤5^th^ percentile for age if <16 yr.

§ Multisystem involvement as defined as abnormality in three or more organ systems including gastrointestinal, muscular, mucous membranes, central nervous system, hematologic, hepatic, or renal. See Figure 1.

Of the 28 non-menstrual cases, 13 (46%) had a skin or soft tissue infection, of which 4 (31%) were post-surgical. Additionally, 10 (36%) had no primary source identified after a median of 4 (range 1, 6) sites cultured, 1 had multiple positive culture sites, 3 had primary bacteremia, 1 had a pulmonary primary site, and 1 a urinary tract site.

All cases were treated with antimicrobial agents, with 58 (95%) receiving a beta-lactam, 41 (67%) clindamycin, and 30 (49%) vancomycin. There was no significant difference in the use of beta-lactams, clindamycin, or vancomycin between menstrual and non-menstrual cases. Nine percent of cases were treated with activated protein C (APC), and 19% were treated with intravenous immunoglobulin (IVIG). Length of hospitalization was shorter in menstrual cases compared to non-menstrual cases (median, 5 vs. 11 days, p<0.001). Cases treated with clindamycin were younger versus those not treated with clindamycin (median 18 vs. 40 years p = 0.02) but there was no difference in the length of hospitalization (6.5 vs. 6.0 days p = 0.67) There were no significant differences in age, sex, number with multiple organ system involvement, or length of hospitalization between TSS cases treated with IVIG versus no IVIG, APC versus no APC, a beta-lactam versus a different antimicrobial agent, and vancomycin versus a different antimicrobial agent. One death (2%) occurred in an 80-year old male.

Menstrual cases from January 1, 2000 to June 30, 2003 (n = 19) were compared to July 1, 2003 to December 31, 2006 (n = 14). There was no significant difference in the frequency of underlying health conditions, age, length of hospitalization, number of positive cultures for SA or MRSA, or treatment with IVIG, APC, beta-lactam antimicrobials, or clindamycin between these two time periods. Vancomycin was prescribed more frequently in July 1, 2003 to December 31, 2006 compared to January 1, 2000 to June 30, 2003 (64% vs. 21% p = 0.03).

### Molecular characterization

Forty-four (72%) of the 61 TSS cases had at least one positive culture for SA; 39 had susceptibility testing results available (Table 4). Four cases (7%) had SA isolates that were MRSA (1 menstrual, 3 non-menstrual). Two MRSA isolates, both from non-menstrual cases, had antimicrobial susceptibility patterns suggestive of CAMRSA (susceptible to quinolones, clindamycin, tetracycline, trimethoprim-sulfamethoxazole, and gentamicin, but resistant to oxacillin in one isolate, and resistant to oxacillin plus erythromycin in the other isolate). One MRSA isolate from a skin/soft tissue infection (non-menstrual) was USA400 (a known CAMRSA clonal group) by PFGE.

**Table 4:** Comparison of Susceptibility Patterns and Superantigens among Isolates Associated with Menstrual and Non-menstrual Toxic Shock Syndrome Cases.

	All Cases	Menstrual	Non-menstrual	p value*
Antimicrobial (n = 44)	39/44 (88%)	25/28 (89%)	14/16 (88%)	1.00
penicillin (n = 35)	0/35 (0%)	0/24 (0%)	0/11 (0%)	1.00
erythromycin (n = 35)	23/35 (66%)	16/24 (67%)	7/11 (64%)	1.00
clindamycin (n = 36)∥	31/36 (83%)	21/24 (88%)	10/12 (83%)	1.00
oxacillin (n = 38)	34/38 (89%)	24/25 (96%)	10/13 (77%)	0.11
quinolones (n = 34)†	32/34 (94%)	22/23 (96%)	10/11 (91%)	1.00
TMP/SMX (n = 33)	32/33 (97%)	22/23 (96%)	10/10 (100%)	1.00
gentamicin (n = 19)	19/19 (100%)	16/16 (100%)	3/3 (100%)	1.00
vancomycin (n = 34)	34/34 (100%)	24/24 (100%)	10/10 (100%)	1.00
Superantigen (n = 44)	26/44 (59%)	19/28 (68%)	7/16 (44%)	0.20
*tst-1* present (n = 25)	20/25 (80%)	17/19 (89%)	3/6 (50%)	0.07
*sea* present (n = 5)	‡ 2/5 (40%)	1/4 (25%)	1/1 (100%)	0.40
*seb* present (n = 20)	3/20 (15%)	0/15 (0%)	3/5 (60%)	<0.01
*sec* present (n = 19)	3/19 (16%)	2/15 (13%)	1/4 (25%)	0.53

Abbreviations:TMP/SMX, trimethoprim-sulfamethoxazole; *tst-1*, toxic shock syndrome toxin 1; *sea*, staphylococcal enterotoxin; *seb*, staphylococcal enterotoxin B; *sec*, staphylococcal enterotoxin C.

*Comparison of menstrual and non-mentstrual TSS cases. Fisher's Exact Chi-Square test comparing menstrual and non-menstrual isolates.

∥Includes inducible clindamycin resistance as evidenced by D-test.

† Testing was performed on gatifloxacin, ciprofloxicin, or levofloxin based on clinical laboratory. If more than one quinolone was tested, isolate was classified as susceptible if the all quinolones tested were susceptible.

‡ Both cases that where sea positive were also tst-1 positive.

Four MSSA isolates from 3 cases also had PFGE performed: a menstrual case with USA200, a non-menstrual case with USA 200 and a menstrual case with two different SA strains from the same vaginal culture (USA700 and USA200). There were no significant differences in susceptibility patterns between isolates associated with menstrual and non-menstrual cases.

Superantigen testing was performed on 26 of 44 (59%) isolates (Table 4). At least one superantigen gene was identified in each isolate. *Tst-1* was the most frequent superantigen gene found and was more common among menstrual vs non-menstrual cases though not statistically significant (89% vs 50% p = 0.07). Both *sea* and *sec* were found in non-menstrual cases. Isolates with *sea* also had *tst-1*. The three isolates with *sec* including two menstrual cases did not have other superantigens. *Seb* was found exclusively in non-menstrual cases (60% vs 0% p<0.01).

### Incidence

Average annual incidence was estimated based on 2000–2003 surveillance. Seven TSS cases from 2000–2003 (all with the specific TSS code) were excluded because they had home zip codes outside of the study area, leaving 31 cases with the TSS-specific ICD-9 code. Five cases were identified using the non-specific TSS codes obtained from the 20% sample of hospitalizations adding 25 additional cases for a total of 56 estimated cases from 2000 through 2003. Average annual incidence per 100,000 persons of all TSS cases was 0.52 cases (95% CI, 0.32–0.77), of menstrual cases was 0.69 (95% CI, 0.39–1.16), and of non-menstrual cases was 0.32 (95% CI, 0.12–0.67) (Table 5). Women aged 13–24 years had the highest incidence with an annual rate of menstrual TSS of 1.41 cases per 100,000 (95% CI, 0.63–2.61).

**Table 5:** Average Annual Toxic Shock Syndrome Incidence by Age and Gender Groups During the Period of Most Complete Case Ascertainment, 2000–2003.

	Annual Incidence*
Risk Group	per 100,000 Persons at Risk (95% CI)
**All TSS**	**0.52 (0.32–0.77)**
All Males	0.23 (0.10–0.44)
All Females	0.79 (0.48–1.22)
**All Menstrual TSS (age 13–54 yr)**	**0.69 (0.39–1.16)**
Menstrual age 13–24 yr	1.41 (0.63–2.61)
Menstrual age 25–54 yr	0.43 (0.19–0.82)
**All Non-menstrual TSS**	**0.32 (0.12–0.67)**
Non-menstrual females ≤24 yr	0.36 (0.12–0.87)
Non-menstrual females >24 yr	0.36 (0.14–0.82)

Abbreviations: CI, Bayesian confidence interval, TSS, toxic shock syndrome; yr, year.

*Annual incidence averaged over all study years, 2000–2003 and estimated by Bayesian statistical methods and Poisson regression.

**Figure 2:**
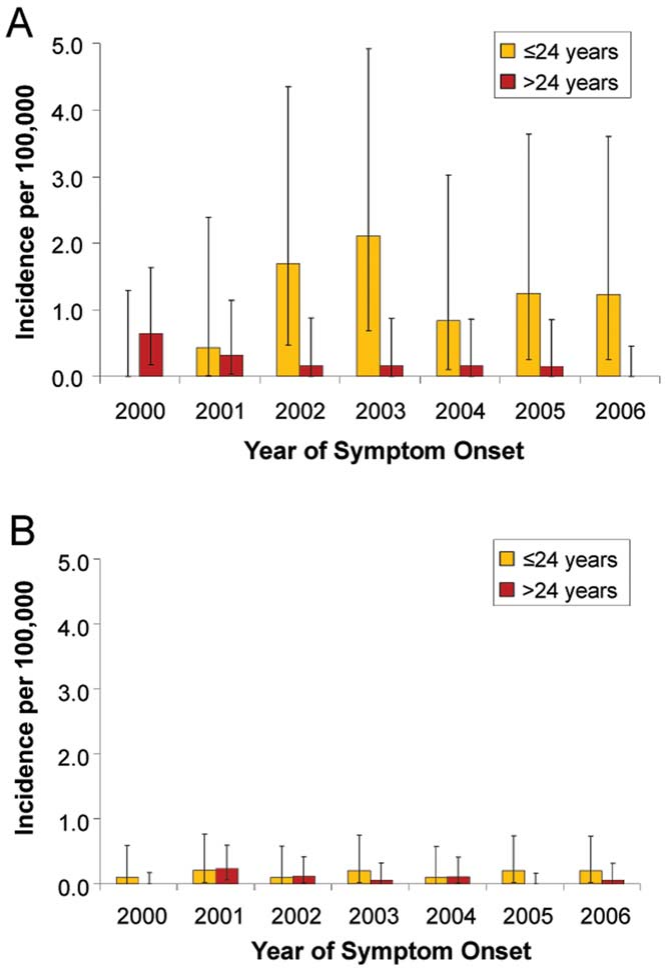
Annual incidence of menstrual and non-menstrual TSS across years 2000–2006. Annual incidence of TSS across years 2000–2006 of menstrual (A) and non-menstrual (B) TSS stratified by age ≤24 and >24 years using only cases receiving the TSS specific ICD-9 code. Error bars represent 95% confidence limits. Frequentist Poisson regressions were used to calculate P-values for test of trend. Over years 2000–2006 among menstrual TSS aged ≤24 years test of trend was not significant (p = 0.22) as was non-menstrual age ≤24 years (p = 0.69) and >24 years (p = 0.40) There was a significant decreasing annual incidence over 2000–2006 in menstrual TSS aged >24 years (test of trend p = 0.02).

For purposes of determining if a change in annual incidence was occurring over the years 2000–2006, the annual incidence was estimated from cases that were coded with the TSS specific code as this was conducted consistently over this time frame. Over the years 2000–2006, the annual incidence rate of all TSS, menstrual TSS, and non-menstrual TSS did not change significantly (test of trend p = 0.63, p = 0.71, and p = 0.77 respectively). When stratifying menstrual and non-menstrual TSS by dichotomous age groups of less than or equal to 24 years and greater than 24 years, there was no significant change in the incidence among the younger menstrual TSS group (test of trend p = 0.22) and younger non-menstrual group (test of trend p = 0.69) over the years 2000–2006 (Figure 2). Similarly the older non-menstrual group did not have a significant change in the annual incidence (test of trend p = 0.40), but older menstrual TSS group had a significant decrease in the annual incidence (test of trend p = 0.02).

## Discussion

The incidence of TSS in Minnesota has declined substantially since the first population based estimates in 1980. The greatest decrease occurred following a peak incidence of 13.7 per 100,000 women aged 15–24 occurred in 1980–1981. By 1986 the incidence among young women had decreased to approximately 1.5–2.5 per 100,000 [7]–[8], [34]. The most recent approximation of TSS case numbers prior to this study, based on national passive case reporting through 1996 and estimates from national insurance databases from 1999–2004, found little change in the absolute number of cases since 1986 [9], [35]. During 2000–2003, the time frame with more complete case ascertainment, we identified similar annual incidence rates compared to those described in 1986 in the MSP area using active-population based surveillance.

Despite this relative stability in TSS incidence over the last 20 years, the prevalent SA strains circulating in the community have shifted. MRSA strains have increased in prevalence during the last decade and MRSA have been reported as the cause of TSS [23], [36]. USA200, typically classified as a health-care associated MRSA strain, has been identified to contain genes for *tst-1* and *sea* [14]. In contrast, CAMRSA strains often carry different superantigen genes with USA400 known to contain *sea*, *seb*, *sec* and/or staphylococcal enterotoxin Q (*seq*) and USA300 strains known to contain staphylococcal enterotoxin K (*sek*) and *seq* but not the most frequent superantigens associated with TSS: *sea*, *seb*, *sec* and *tst-1* [13], [22], [25]–[26]. One of our TSS cases had a USA400 MRSA strain.

Over the past 10 years, USA300 MRSA strains have substantially increased in prevalence in Minnesota, replacing USA400 as the most common strain of CAMRSA. As a percentage of MN CAMRSA isolates obtained from sentinel site surveillance, USA300 strains increased from 6% in 2000 to 68% in 2005. In contrast, USA400 strains decreased from 63% in 2000 to 11% in 2005 of MN CAMRSA isolates [37]. Among the 44 TSS cases with a positive culture for SA, we identified 4 (13%) MRSA isolates, of which one had a pulsotype commonly associated with CAMRSA. The increasing prevalence of USA300 among circulating strains, and the lack of TSS cases found due to USA300 suggests that although USA300 is associated with an increased occurrence of some staphylococcal syndromes including skin and soft tissue infections, osteomyelitis and pneumonia, it does not appear to be associated with TSS.

TSS remains an important cause of morbidity among young people, with the greatest impact on young women. During our surveillance period, the median age for all cases was 21 years, and for menstrual cases was 18 years. Cases stayed a median of 6 hospital days including intensive care unit time. While most recover, we identified one death due to TSS. Despite public health efforts, such as removal of high-absorbency tampon products and public safety announcements, menstrual TSS continues to occur at rates similar to other infections of public health importance such as *N. meningitidis* (annual incidence 0.28/100,000) [38] and invasive group A *Streptococcus* (annual incidence 3.6/100,000) [39].

Unfortunately, no single diagnostic test is available to clearly identify persons with TSS. Using the CDC case definition we found an incidence similar to past population-based estimates of incidence. The case definition was developed in 1980 to identify TSS risk factors and has only been slightly modified since then with the most recent update occurring in 2011 [40]. As the pathophysiology has been further described, a wide range in the severity of TSS has been recognized. Individuals who seek medical care promptly, receive appropriate early interventions, or have partial immunity are more likely to experience a mild form of the illness. Advances in supportive care such as early goal directed fluid resuscitation likely prevent the development of the most severe manifestations of the illness. Because of these factors, a large portion of the TSS disease burden is very likely not being identified by strictly applying the CDC TSS case definition. Therefore, our estimates represent the incidence of the most severe form of TSS. In the absence of a definitive diagnostic test, it is difficult to quantify the burden of disease of less severe presentations of TSS. For this reason, clinicians should not rely on the epidemiologic case definition to determine when to institute appropriate treatment for TSS as this may lead to unnecessary morbidity.

Interestingly, there was a decrease in the rates of menstrual TSS among women older than 24 years when comparing rates of disease over the years 2000–2006. Possible factors leading to a decreasing incidence include decreasing tampon use, increasing levels of protective antibody in this population, or decreasing use of intravaginal contraceptive devices. An additional protective factor may be the increasing frequency of menstrual suppression techniques within this group, as the safety and acceptability of this practice among patients and practitioners has increased in the last decade [41]–[42]. The prevalence of menstrual suppression among women of any age group is unknown.

We conducted this study within a single geographic area. While there is racial and ethnic diversity in the MSP area, it may not be representative of the racial and ethnic makeup of other geographic areas and there may be unknown factors unique to this region. Additionally, a select group of ICD-9 codes were used to capture cases. If additional, lower yield codes had been included, we may have identified additional TSS cases. Therefore, our calculated incidence likely represents a low estimate of the actual incidence.

In conclusion, we observed a stable incidence of both menstrual and non-menstrual TSS in the years 2000–2003 compared to the late 1980s, with the highest incidence among women aged 13–24 years. There was also no significant increase in annual TSS incidence over the years 2000–2006. While one TSS case due to CAMRSA (USA400) was identified, the increased prevalence of CAMRSA in Minnesota did not appear to affect the incidence of menstrual or non-menstrual TSS. It would be useful to conduct surveillance for TSS cases in other populations and geographic areas that may have different prevalent staphylococcal strains and host susceptibilities to TSS, as well as assess the toxins produced by implicated strains in order to monitor changes in the epidemiology of TSS.
